# Incidental Finding of Diverticulosis of the Appendix with Sessile Serrated Adenoma

**DOI:** 10.7759/cureus.8230

**Published:** 2020-05-21

**Authors:** Areeka Memon, David B Stoeckle

**Affiliations:** 1 Osteopathic Medicine, Edward Via College of Osteopathic Medicine, Blacksburg, USA; 2 General Surgery, Edward Via College of Osteopathic Medicine, Blacksburg, USA

**Keywords:** appendicitis, diverticulosis, diverticulosis of the appendix, sessile serrated adenoma, diverticulitis, diverticulitis of the appendix, appendiceal diverticulosis

## Abstract

Diverticulosis and sessile serrated adenomas of the colon are common findings on routine colonoscopy. However, diverticulosis of the appendix is rare and is usually only discovered due to conversion to diverticulitis or as an incidental finding. Diverticulitis of the appendix can present as appendicitis but is associated with more risks. A pathology report is important in diverticulosis of the appendix due to the association with malignancy. This case report reviews a 52-year-old female who presented to the emergency department with right lower quadrant pain who was diagnosed with acute appendicitis and was incidentally found to have diverticulosis of the appendix with a sessile serrated adenoma.

## Introduction

Diverticulosis is a condition that consists of pouch-like structures that emerge at points of weakness throughout the colon. However, they can rarely occur in other places throughout the GI tract as well [1]. The appendix is one of the rare locations that diverticulosis can appear, with an incidence of about 1% [2]. When diverticulosis of the appendix appears, it can present similarly to appendicitis and is usually only diagnosed during appendectomy [3].

Sessile serrated adenomas are most commonly found on the right side of the colon but can rarely be found in the appendix as well [4]. Sessile serrated adenomas are diagnosed when displaying features such as branching of crypts, dilatation of the base of the crypts, and growth of crypts parallel to the muscularis mucosae [5].

## Case presentation

A 52-year-old female with no significant past medical history presented to the emergency department with generalized abdominal pain. The pain began one day prior to admission and she had not been able to eat since the start of her pain. She did not take any medications and stated that her pain was a seven out of 10 in intensity on presentation. She admitted to one episode of vomiting but denied any blood in the vomitus, diarrhea, constipation, fever, chills, chest pain, shortness of breath, dysuria, hematuria, melena, or hematochezia. Physical exam demonstrated mild diffuse tenderness to palpation with pain worse in the right upper quadrant, and the patient had hyperactive bowel sounds.

**Figure 1:**
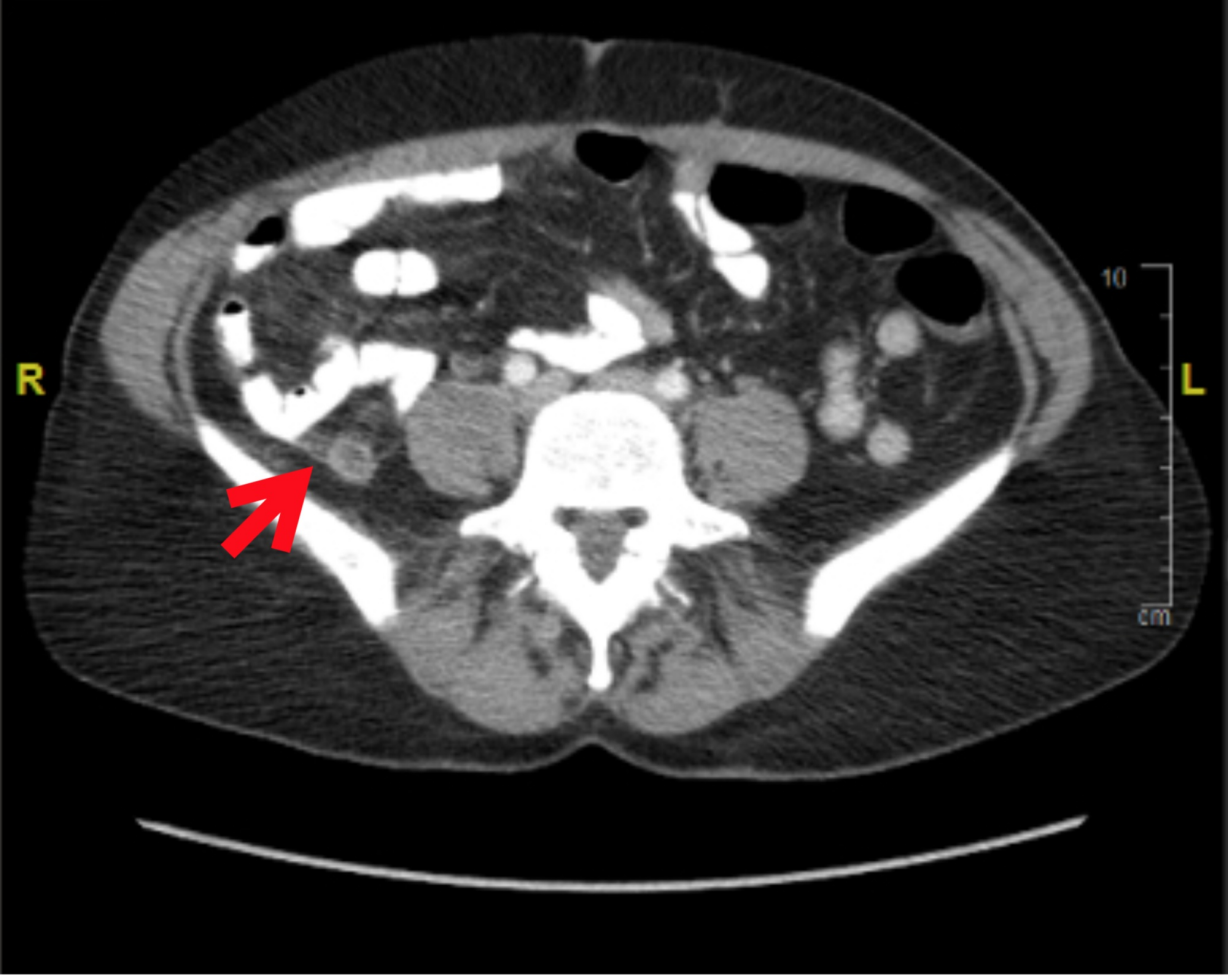
Axial CT scan demonstrating appendicitis CT: computed tomography

**Figure 2:**
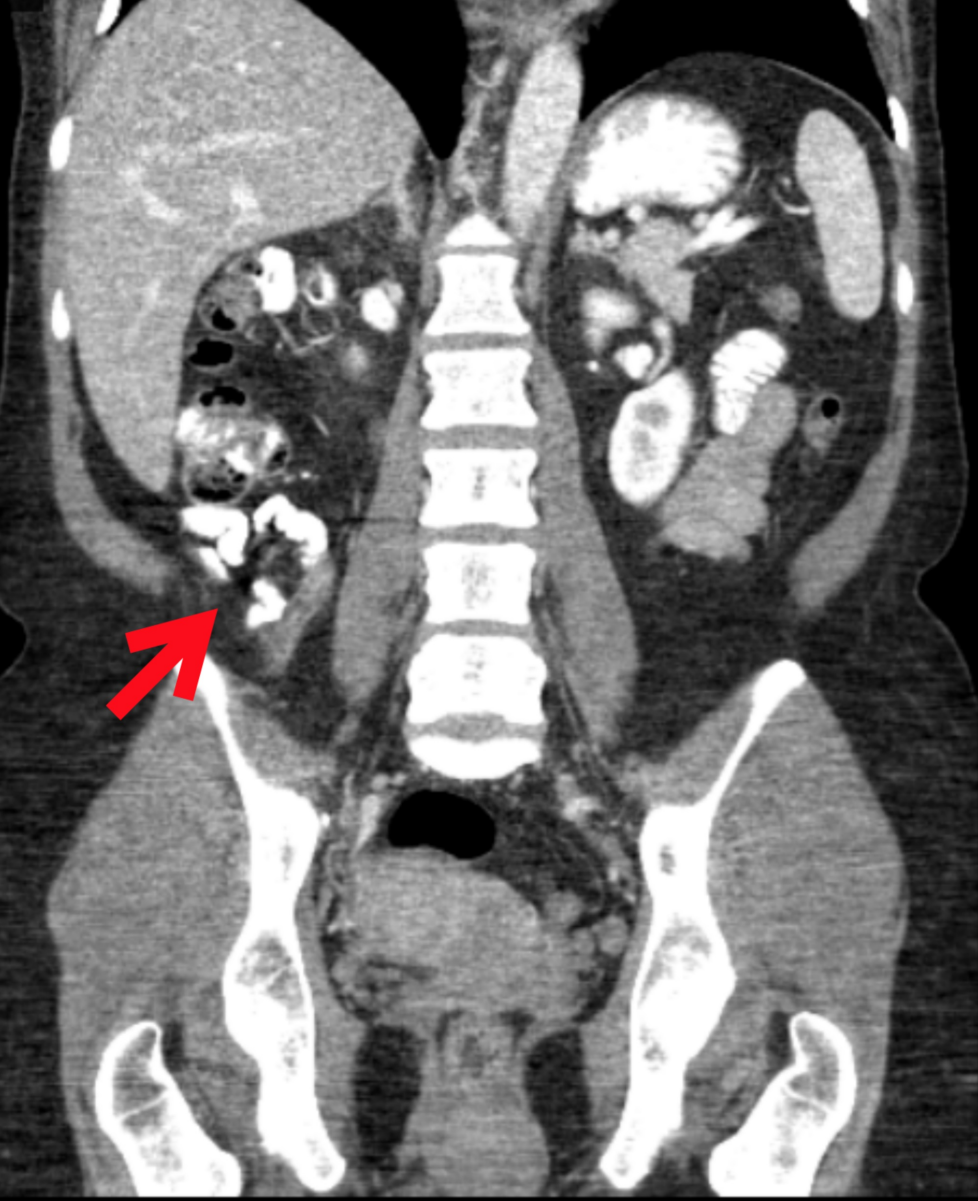
Coronal CT scan demonstrating appendicitis CT: computed tomography

The patient had labs and imaging completed, including a urinalysis with culture, serum beta-human chorionic gonadotropin, complete metabolic panel, complete blood count with differential, and computed tomography (CT) of the abdomen and pelvis with intravenous (IV) contrast. CT showed no bowel obstructive changes. The appendix was thickened and dilated, measuring up to 10 mm with adjacent periappendiceal inflammatory changes. Findings were consistent with acute appendicitis with no abscess or perforation (Figures 1-2).

**Figure 3:**
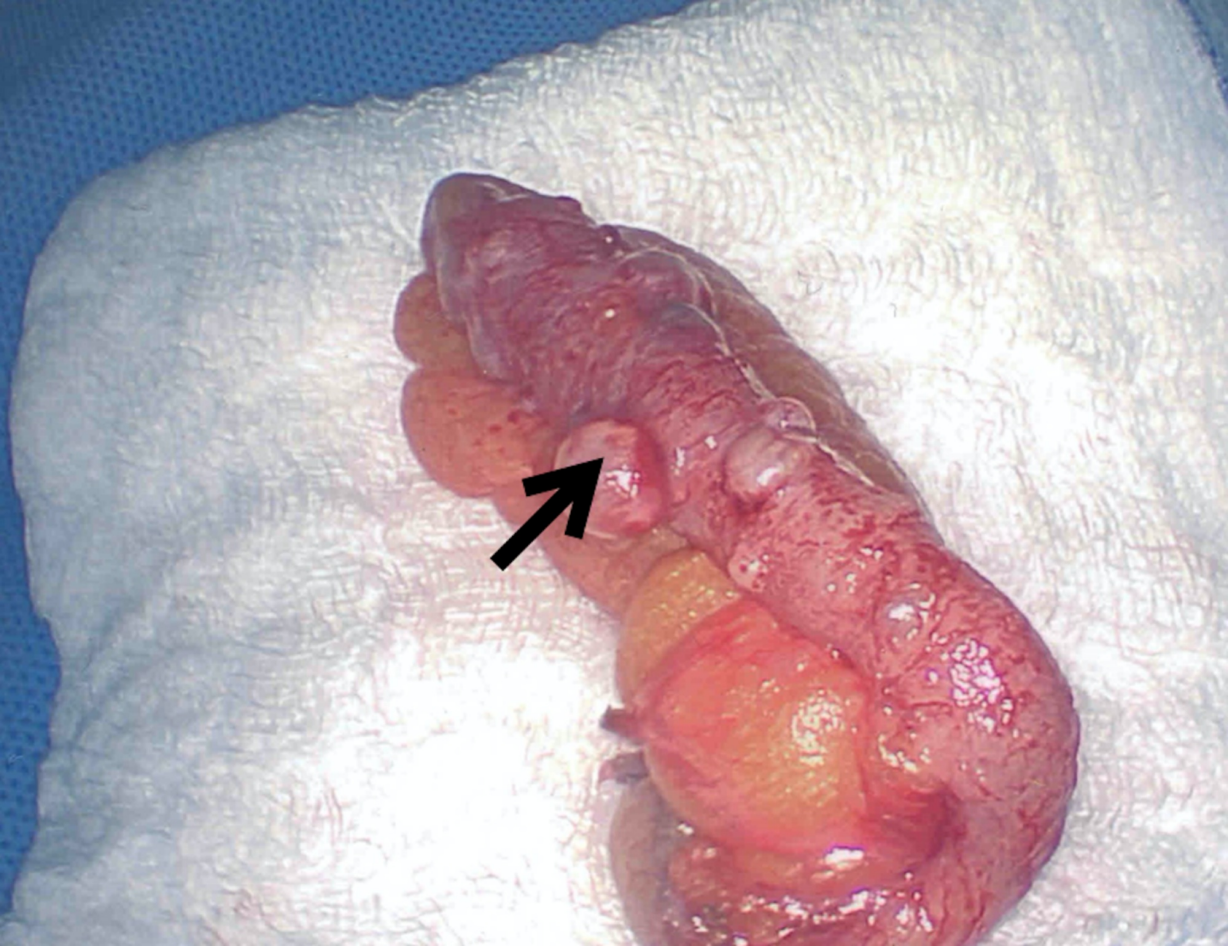
Removed appendix showing diverticula and sessile serrated adenoma

**Figure 4:**
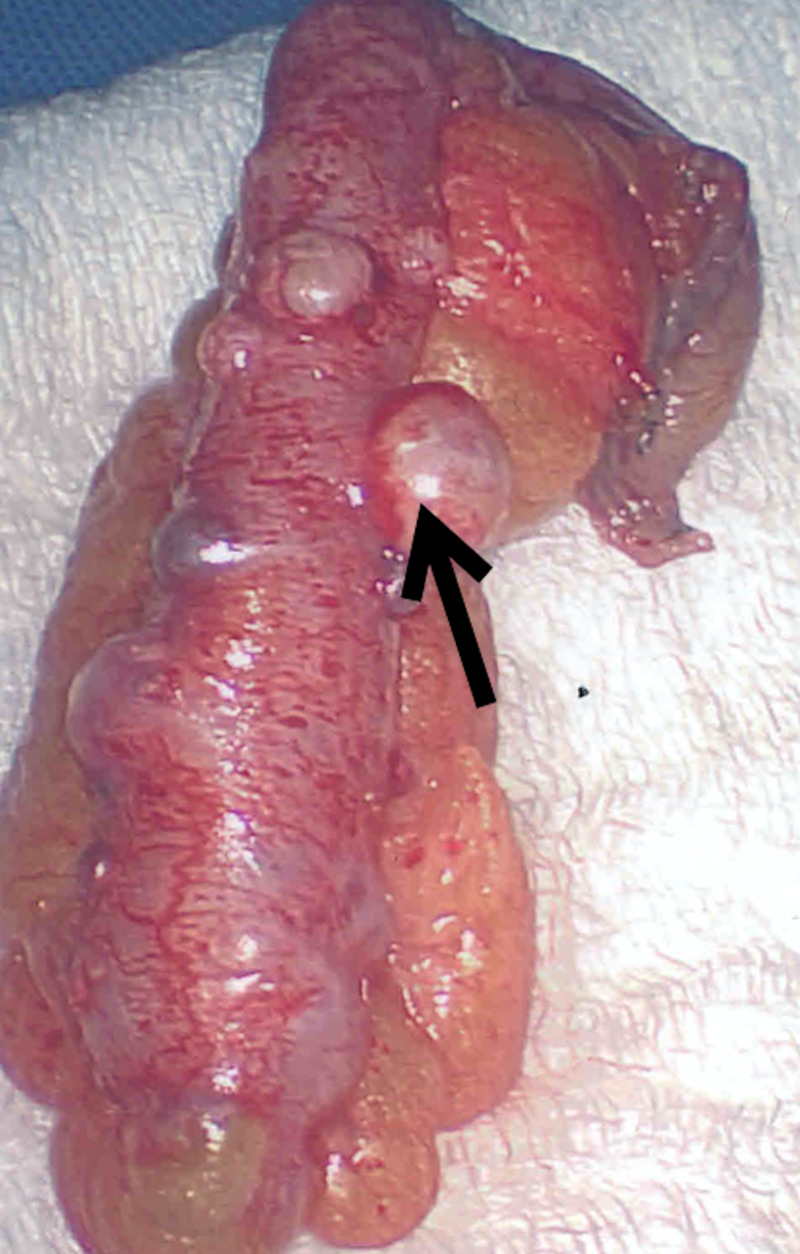
Removed appendix showing diverticula and sessile serrated adenoma

Surgery was consulted and laparoscopic appendectomy was performed the same day. The procedure was performed with the patient in the supine position and under satisfactory general anesthesia. The patient was prepped with Chloraprep (Becton, Dickinson and Company, Franklin Lakes, New Jersey) and draped sterilely. Incision sites were injected with 0.25% marcaine. An incision was made at the umbilicus, and the abdomen was inflated with 15 mmHg of carbon dioxide. A trocar, scope, and camera were passed into the abdomen and bilateral lower quadrants. The appendix was located, examined, and then grasped. The appendix presented grossly with nodular changes (Figures 3-4). A dissection plane was made at the base of the appendix. The appendix was then divided with an endoscopic stapler. The mesoappendix was then divided with the endoscopic stapler. The appendix was placed into an endopouch and removed through the left-sided trocar site. The area was irrigated and suctioned. There was no significant bleeding. First, the left lower trocar was removed and the site was closed with a GraNee needle (R-Med, Inc., Navarre Ave, Oregon) with 0 vicryl, and then the abdomen was deflated and the remaining trocars were removed. Incisions were closed with a subcuticular 4-0 chromic suture and Dermabond glue. The patient tolerated the procedure well and left the operating room in satisfactory condition.

The pathology report of the appendix reported a vermiform appendix showing acute appendicitis with associated diverticulosis and a serrated polyp, suggestive of a sessile serrated adenoma. No high-grade dysplasia or malignancy was seen.

## Discussion

While appendicitis is common, diverticula and sessile serrated adenomas are rare and can sometimes be found incidentally on appendectomy. Diverticula of the appendix can be either acquired or congenital. Congenital diverticula are rarer and generally seen as a single diverticulum, but the acquired form is usually diverticulosis [6]. Congenital diverticula are generally true and consist of the mucosa, submucosa, serosa, and muscular layers, while the acquired diverticula are usually false and lack the muscular layers [7]. The congenital diverticula form due to abnormalities, including epithelial inclusion cyst residues, appendiceal luminal rechannelization, and failure of vitelline duct obliteration [7]. Acquired diverticula are more prone to perforation than congenital diverticula [8]. Risk factors for diverticulosis of the appendix include male gender, age greater than 30, history of Hirschprung’s disease, and cystic fibrosis [8].

Sessile serrated adenomas are usually found in the colon and are part of the adenoma-carcinoma sequence of colonic malignancy. However, it is debated whether sessile serrated adenomas of the appendix are associated with the same mutations and risk for malignancy as colonic adenomas [5]. Some research has shown that the pathway differs in appendiceal lesions in that the KRAS mutation is present but the BRAF mutation is less commonly found [9]. Complications with sessile serrated adenomas occur when the goblet and mucin-producing cells lead to the formation of abscesses and rupture [10]. The tubular structure of the adenomas can also lead to obstruction and this can be followed by an increase in intraluminal pressure, resulting in rupture of the adenoma [10].

While diverticulosis of the appendix can be incidentally found with acute appendicitis, diverticulitis of the appendix can be an isolated finding as well. In fact, diverticulitis of the appendix and appendicitis have different risk factors and when found alone should be managed differently. Diverticulitis of the appendix has a higher rate of mortality, more rapid progression, and association with malignancy when compared to acute appendicitis [8]. The age of patients with diverticulitis of the appendix typically ranges from 37 to 39 years, but appendicitis is commonly found in younger individuals [8]. While the pain from appendicitis usually begins periumbilically, the pain from diverticulitis of the appendix begins in the right lower quadrant [8]. Diverticulosis of the appendix is usually clinically silent, but it can present with intermittent abdominal pain [8].

If diverticulitis of the appendix is a possible differential diagnosis, it can be evaluated through a barium enema, ultrasound, or CT [8]. This diagnosis is usually not made due to the rarity of the condition and lack of symptomatology, however, the most likely patient is a male with abdominal pain. Even with the diagnostic tools mentioned, diagnosis can be difficult due to the diverticula being small or an obstruction causing poor barium filling [8]. 

A missed diagnosis of diverticulitis of the appendix can lead to several complications. Perforation is one of the most common complications and can be as high as 66% [8]. Other complications include hemorrhage, pelvic pseudocyst formation, appendicovescial fistula formation, and pseudomyxoma peritonei [8].

## Conclusions

Due to the difficulty of visualizing diverticulosis of the appendix on imaging, this patient was diagnosed following surgery, but acute inflammation of the diverticula would have been managed similarly. It is imperative to assess the mucosa present in the nodular surface to diagnose possible malignancies that can be associated with sessile serrated adenomas. In conclusion, this case report highlights a rare finding that should be recognized and appropriately worked up. It was integral in this case to diagnose and perform surgery in a timely manner. It was also important to obtain a detailed pathology report that addresses concerns of potential malignancy.
